# Effect of repolishing on the color stability of a supra-nano spherical filled composite resin: An in vitro study

**DOI:** 10.34172/joddd.025.41464

**Published:** 2025-06-30

**Authors:** Merve Haberal, Ezgi Türkoğlu, Yusuf Bayraktar

**Affiliations:** Department of Restorative Dentistry, Faculty of Dentistry, Kırıkkale University, Kırıkkale, Turkey

**Keywords:** Coloring agents, Dental composite resin, Spectrophotometry, Staining

## Abstract

**Background.:**

The present study aimed to assess the impact of staining drinks and repolishing on the color stability of a monochromatic composite resin.

**Methods.:**

Eighty composite resin specimens (Omnichroma, Tokuyama Dental, Tokyo, Japan) were fabricated in stainless steel molds and cured with an LED light-curing device (Elipar, 3M ESPE, St. Paul, MN, USA). Initial color scores were recorded after immersion in artificial saliva for 24 hours. Subsequently, the specimens were exposed to staining drinks (artificial saliva, tea, detox drink, cherry juice, and coffee) for 4 days, followed by color score assessments. Final color measurements were obtained after repolishing with a composite resin polishing kit (CLEARFIL^TM^ Twist DIA, Kuraray, Tokyo, Japan). Statistical analyses were conducted using SPSS 16.0 (*P*<0.05).

**Results.:**

Significant differences were found in staining intensity (ΔE1) between the groups; artificial saliva produced the fewest stains, whereas detox drinks produced the most. Nonetheless, no discernible variation was seen in the average ΔE1 and ΔE2 scores, indicating equivalent staining and discoloration. Color parameter shifts were noted in all the groups after staining and after repolishing. Staining drinks induced noticeable color changes, especially in the tea, detox drink, cherry juice, and coffee groups. Repolishing resulted in minor color changes but did not fully restore the original appearance.

**Conclusion.:**

ΔE scores after staining and after repolishing composite resins were similar, indicating that restoration color returned to its original state after polishing as much as after staining. Immediate replacement of stained restorations may not be needed; this study recommends repolishing before considering replacement.

## Introduction

 Dentists frequently prefer ceramic materials and resin-based composite (RBC) resins in their restorative treatments due to their aesthetics. The aesthetic success of composite resin restorations depends primarily on their surface properties and color stability.^1^ Aesthetically successful restorative materials replicate natural teeth and retain their original appearance.^2^ Prolonged daily exposure to staining drinks may stain composite resins, CAD/CAM-processed composite resins, and ceramic materials.^3,4^ The progressive alteration of color in dental restorations over time constrains their durability and overall quality.^5^ For RBC_S_, the color change depends on the foods and drinks consumed, the composite resin material’s composition, and its surface properties.^6^ In clinical practice, colorimeters, digital cameras, and spectrophotometers are frequently used for color scoring. The International Commission on Illumination’s CIE L*a*b* system is typically used by spectrophotometers to quantify color.^7^

 Finishing and polishing procedures reduce the surface roughness of restorations and ensure surface integrity. Therefore, these are essential steps that enhance the appearance and durability of restorations by decreasing plaque retention and staining.^8^ Silicon carbide brushes, polishing discs, rubber spirals with diamonds embedded in them, carbide or diamond burs, and polishing pastes are examples of materials used in polishing. The types, compositions, and hardness of the abrasives used in these polishers, which are employed in one or more stages of the finishing and polishing procedures, differ substantially.^9^ Composite resin restorations that exhibit superficial staining over time can regain their aesthetic appeal after polishing. The durability and appearance of teeth that have been repaired can both be enhanced by proper polishing.^10^

 The physical properties of many RBC_S_ on the market have been improved by modifying the size, type, and quantity of the matrix or filler particles.^11^ Manufacturers are now introducing RBC_S_ with a single color system instead of more complicated color systems because of the application of nanotechnology in dentistry. Dentists have recently been provided with monochromatic composite resins that work with any hue of teeth. The advantage of the color-matching feature is that it eliminates the need for color removal.^12^ Omnichroma (Tokuyama Dental, Tokyo, Japan) is one of these composite resins that does not contain any color pigment. Thanks to their translucency, composite resins can create a chameleon effect by transferring their own color to dental tissues and reflecting the adjacent tooth color, resulting in improved color harmony. This advanced chromatic technique is intended to regulate a composite resin’s optical characteristics.^13^

 Some studies have assessed the color change of the mentioned composite resin material (Omnichroma) after bleaching, color stability after immersion with various drinks, and the chameleon effect.^14-16^ Our study provides a new contribution to the literature regarding polishing Omnichroma after immersion in staining drinks and comparing the results with the initial color values.

 This study evaluated the effect of different staining drinks and repolishing on the color changes of a monochromatic composite resin with supra-nano fillers. The null hypotheses were as follows: 1) Consumption of different staining beverages does not affect the color of the supra-nano-filler monochromatic composite resin over time. 2) The repolishing procedure does not affect the color change of the composite resin.

## Methods

 Eighty composite resin specimens were used in this study. While preparing the resin composite resin specimens, a monochromatic composite resin with supra-nano fillers (Omnichroma, Tokuyama Dental, Tokyo, Japan) was placed in a stainless steel mold with a diameter of 10 mm and a thickness of 1 mm. Two thin glass plates were then placed on the upper and lower surfaces of the stainless steel mold, and finger pressure was applied to remove excess composite resin. The composite resin specimens were polymerized from the upper and lower surfaces using a light-emitting diode (LED) light-curing device (Elipar, 3M ESPE, St. Paul, MN, USA) with a power of 1,200 mW/cm^2^ for 20 seconds. The upper surfaces of the specimens were polished using a composite resin polishing kit (CLEARFIL^TM^ Twist DIA, Kuraray, Tokyo, Japan) by the same operator using a similar procedure (medium and fine grits, respectively). The specimens were randomly divided into five groups (n = 16) and immersed in 20 mL of artificial saliva at 37 °C for 24 hours. Table 1 presents the study groups. The initial color scores (L*, a*, and b*) of the specimens were then measured with a spectrophotometer (VITA Easyshade Compact; VITA Zahnfabrik, Bad Sackingen, Germany) from the center of the specimens on a white background in a color measurement cabinet illuminated with a D65 lamp. After the initial color scores, the specimens were analyzed for tea (Lipton Yellow Label Tea, Unilever, Istanbul, Turkey), detox drink (Elite Detox Defence, Elite Naturel Organik Gıda San. ve Tic. A.S., Ankara, Turkey), sour cherry juice (DIMES Sour Cherry Juice, DIMES Ltd., Tokat, Turkey), and coffee (Nescafé Gold, Nestlé, Frankfurt/Main, Germany) drinks. Table 2 lists the composite resin, drinks, and their contents used in the study.

**Table 1:** Groups included in the study

**Groups**	**Drinks**
Group 1 (G1)	Artificial saliva
Group 2 (G2)	Tea
Group 3 (G3)	Detox drink
Group 4 (G4)	Cherry juice
Group 5 (G5)	Coffee

**Table 2:** Materials, drinks, and their contents used in the study

**Materials and Drinks**	**Contents**
Omnichroma (Tokuyama Dental, Tokyo, Japan)	Organic: Urethane dimethacrylate, Triethylene glycol dimethacrylate, Mequinol, Dibutyl hydroxyl toluene and ultraviolet absorber Inorganic: Spherical silica-zirconia filler 79 wt%, 68 vol%
CLEARFIL^TM^ Twist DIA (Kuraray, Tokyo, Japan)	Matrix: Rubber Abrasives: Diamond particles Particle size: Prepolisher medium grit: 25-35 μm High-shine polisher fine grit: 4-8 μm
Artificial Saliva	1.160 g/L sodium chloride, 0.600 g/L calcium chloride, 0.600 g/L potassium phosphate, 1.491 g/L potassium chloride, 0.050 g/L sodium fluoride, trace amounts of sodium hydroxide
Tea (Lipton Yellow Label Tea, Unilever, İstanbul, Turkey)	Black tea
Detox Drink (Elite Detox Defence, Elite Naturel Organik Gıda San. ve Tic. A.S., Ankara, Turkey)	Organic carrot puree (40%), organic orange juice (30%), organic apple juice (15%), organic lemon juice (10%), organic turmeric powder (5%)
Cherry Juice (DİMES Sour Cherry Juice, DIMES Ltd., Tokat, Turkey)	Water, sugar, sour cherry juice concentrate, acidity regulator (citric acid), fruit content: minimum 20%
Coffee (Nescafé Gold, Nestlé Frankfurt/Main, Germany)	100% soluble coffee

 After the specimens had been immersed in drinks for 4 days, color scores were performed similar to the initial procedure. A previous study reported that a glass of soft drink was consumed in an average time of 15 minutes.^17^ Accordingly, if one glass of soft drink is consumed daily, a 4-day immersion period corresponds to approximately one year.

 Before the color measurement procedure, all the specimens were rinsed with distilled water for 20 seconds and air-dried for 10 seconds. Three scores were made for each specimen, and the average score was used. After the top surfaces of the specimens were polished with a composite resin polishing kit (CLEARFIL^TM^ Twist DIA, Kuraray, Tokyo, Japan), the color measurement of the specimens was performed again with a similar procedure.

 The color change scores (ΔE) of the groups were calculated according to the CIEDE2000 color difference formula:


$$\sqrt{{\left(\frac{\Delta L^{\prime }}{K_{L}S_{L}}\right)}^{2}+{\left(\frac{\Delta C^{\prime }}{K_{C}S_{C}}\right)}^{2}+{\left(\frac{\Delta H^{\prime }}{K_{H}S_{H}}\right)}^{2}+RT(\frac{\Delta C^{\prime }}{K_{C}S_{C}})(\frac{\Delta H^{\prime }}{K_{H}S_{H}})}$$

L*, a*, and b* scores were measured on a white background to calculate ΔE00.

 In this study, statistical analyses were performed using SPSS 16.0 (SPSS Inc, Chicago, IL, USA). The tests included one-way ANOVA, post hoc Tukey tests, and t-test (*P* < 0.05).

## Results

 In addition to the color change, the translucency parameters (TP) of the groups were also calculated, but L*, a*, and b* scores could be measured on the white background but not on the black background.

 A statistically significant difference was noted between the stained specimens regarding the mean ΔE1 scores. Accordingly, the most stained group was the detox drink group, while the least stained group was the artificial saliva group (Table 3).

**Table 3:** The mean ΔE_1_ scores of the groups. The same superscript letters indicate no statistical difference between groups

**Artificial saliva**	**Tea**	**Detox drink**	**Cherry juice**	**Coffee**	***P*** ** value**
0.50 ± 0.49^a^	5.58 ± 1.35^b^	16.98 ± 3.02^c^	3.48 ± 0.82^d^	5.94 ± 2.53^b^	< 0.001

*There is no statistically significant difference between the groups with the same lowercase superscript letters; *P* > 0.05.

 The t-test results showed no statistically significant difference between the mean ΔE1 and ΔE2 scores of the groups (Table 4). Along with the average ΔE1 scores of the groups, the average ΔE2 scores of the discolored specimens after polishing were also calculated. The t-test results revealed no significant difference between the average staining (ΔE1) and color lightening ΔE2 scores of the groups. It can be said that the groups were discolored as much as they were stained.

**Table 4:** The mean ΔE_1_ and ΔE_2_ scores of the groups. The *P*-values are also given

	**Artificial saliva**	**Tea**	**Detox drink**	**Cherry juice**	**Coffee**
ΔE_1_	0.50 ± 0.49	5.58 ± 1.35	16.98 ± 3.02	3.48 ± 0.82	5.94 ± 2.53
ΔE_2_	0.30 ± 0.23	4.75 ± 1.19	16.04 ± 3.19	2.93 ± 1.18	4.74 ± 2.00
*P* value	0.149	0.076	0.398	0.135	0.147

 The mean L_0_ and L_1_ scores in the artificial saliva group differed significantly (*P* = 0.049). The L_0_ and L_2_ scores did not differ from one another (*P* = 0.35). Compared to the b_0_ score, the mean b_1_ and b_2_ scores were significantly lower (shifted toward blue, *P* = 0.002) (Table 5).

**Table 5:** The mean L, a, and b scores of the groups. The same superscript letters indicate no statistical difference between groups

	**Artificial Saliva**	**Tea**	**Detox drink**	**Cherry juice**	**Coffee**
L_0_	91.71 ± 1.51^a^	93.01 ± 1.78^a^	92.05 ± 1.40^a^	92.19 ± 2.29^a^	91.89 ± 1.89^a^
L_1_	92.01 ± 1.71^bc^	88.13 ± 1.19^b^	93.00 ± 1.99^a^	87.73 ± 2.10^b^	88.39 ± 2.52^b^
L_2_	91.88 ± 1.71^ac^	91.34 ± 2.07^c^	92.51 ± 1.61^a^	91.61 ± 1.93^a^	92.71 ± 1.71^a^
*P* value	0.013	< 0.001	0.05	< 0.001	< 0.001
a_0_	-4.21 ± 0.49^a^	-4.11 ± 0.62^a^	-4.15 ± 0.37^a^	-4.04 ± 0.40^a^	-4.16 ± 0.48^a^
a_1_	-4.18 ± 0.49^a^	-2.10 ± 0.96^b^	-15.54 ± 7.84^b^	-2.53 ± 0.79^b^	- 2.93 ± 1.18^b^
a_2_	-4.17 ± 0.46^a^	-3.78 ± 0.66^c^	-4.47 ± 2.52^a^	-3.37 ± 0.45^c^	-3.60 ± 0.39^c^
*P* value	0.224	< 0.001	< 0.001	< 0.001	< 0.001
b_0_	6.86 ± 0.91^a^	7.24 ± 0.84^a^	7.42 ± 1.06^a^	8.06 ± 1.38^a^	6.73 ± 0.98^a^
b_1_	6.62 ± 1.04^bd^	12.22 ± 1.16^b^	41.49 ± 10.13^b^	8.18 ± 1.56^a^	13. 46 ± 2.79^b^
b_2_	6.73 ± 0.91^cd^	7.46 ± 0.88^a^	9.47 ± 1.30^c^	7.35 ± 1.46^b^	8.41 ± 0.81^c^
*P* value	0.002	< 0.001	< 0.001	0.004	< 0.001

* There is no statistically significant difference between the groups with the same lowercase superscript letters; *P* > 0.05.

 The mean L_1_ score in the tea group was much lower than the L_0_ score (*P* < 0.001). The average L_2_ score was much lower than the L_0_ score (*P* = 0.005) but somewhat higher than the L_1_ score. There was a significant difference (*P* < 0.001) between the mean a_1_ and a_2_ scores. The a_1_ score was significantly greater than the a_0_ score (shifted toward red, *P* < 0.001), and the mean a_0_ score had the lowest value. The average scores for a_0_, a_1_, and a_2_ varied significantly (*P* < 0.05). The mean b_1_ score (shifted toward yellow, *P* < 0.001) was much greater than the mean b_0_ score. However, the mean b_0_ and b_2_ scores did not vary significantly (*P* = 0.70) (Table 5).

 The mean L scores in the detox drink group did not change significantly (*P* = 0.05). Compared to the a_0_ and a_2_ scores, the mean a_1_ score was significantly lower (shifted toward green, *P* < 0.001). The mean b_1_ score in the detox drink group was changed to yellow (*P* < 0.001) and decreased after repolishing, indicating a significant difference from the b_0_ and b_2_ scores. Nonetheless, there was still a significant difference (*P* < 0.001) between the mean b_2_ and the b_0_ scores (Table 5).

 Compared to the L_0_ and L_2_ scores, the cherry juice group’s mean L_1_ score was much lower (*P* < 0.001). There was a significant difference (*P <*0.001) between the mean a_1_ and a_2_ scores. The a_1_ score was significantly greater than the a_0_ score (shifted toward red, *P* < 0.001), and the mean a_0_ score had the lowest value. The average scores for a_0_, a_1_, and a_2_ varied significantly (*P* < 0.05). Significantly less than the b_0_ and b_1_ scores was the mean b_2_ score (shifted toward blue, *P* = 0.004) (Table 5).

 The mean L_1_ score in the coffee group was P < 0.001, which was considerably lower than the L_0_ and L_2_ values. The a_1_ score was significantly greater than the a_0_ score (shifted toward red, *P* < 0.001), while the mean a_0_ score had the lowest value. The average scores for a_0_, a_1_, and a_2_ varied significantly (*P* < 0.05). After repolishing, the mean b_1_ score decreased and was significantly greater than the b_0_ and b_2_ scores (shifted toward yellow, *P* < 0.001). Nevertheless, there was still a significant difference (*P* < 0.001) between the b_2_ and b_0_ scores (Table 5).

## Discussion

 The adsorption of external coloring agents on the composite resin surface and their absorption by the resin matrix can cause color changes and compromise aesthetic results. Mouthwashes, fruit juices, coffee, tea, cola, and a wide range of other drinks can all have distinct effects on the color stability of composite resins. Maintaining color throughout the functional life of dental restorations is one of the most critical features of aesthetic restorative materials in terms of treatment durability.^18^ The proper finishing and polishing procedures of composite resin restorations minimize the external staining of restorations by preventing the accumulation of plaque biofilm and staining agents.^19^ Composite resins may become stained externally, which can be resolved by a few different methods, including brushing, bleaching, and repolishing. The type of toothpaste used by patients while brushing their teeth, the hardness of brushing, and the frequency of brushing affect the removal of external stains. Repolishing is a faster method performed by the dentist independently of the patient. Bleaching agent applications are more expensive than these two methods and require many sessions.^20^

 This study investigated the changes in color stability of monochromatic composite resins consisting of supra-nano fillers after exposure to various staining drinks and repolishing processes. The study results showed the importance of the materials used and the surface treatments applied in maintaining the aesthetic properties of such materials, which has important implications for clinical applications and materials science. The supra-nano-filled monochromatic composite resin specimens immersed in different staining drinks showed coloration over time, and it was observed that these stained composite resin surfaces could return to a color close to their previous color with repolishing. According to these results, both hypotheses were rejected.

 Color measurements in this investigation were conducted using VITA Easyshade (VITA Zahnfabrik, Bad Sackingen, Germany). According to Kim-Pusateri et al,^7^ the accuracy percentage was 92.9%. Many studies have employed Vita Easyshade as a measuring method.^21-23^ In this study, three measurements were made, and average L*, a*, and b* scores were used to achieve the most accurate results. Clinical and in vitro dental research regularly makes use of the CIE L*a*b* (CIELab) color notation system.^24-26^ Lightness or darkness is indicated by the L* score, redness or greenness by the a* score, and yellowness or blueness by the b* score. These should be noted to replicate the long-term effectiveness of cosmetic repairs and are also crucial for assessing optical qualities.^27^ To guarantee consistency of CIELab color differences and enhance connection with visual evaluation, the CIEDE2000 color difference formula was created.^28^ The CIEDE2000 formula, which is widely used in the current literature, was used to determine the color changes in this study.^29,30^ In CIEDE2000, the color difference or color change over time is indicated as ΔE00; a greater score of ΔE00 indicates a bigger difference that is easier for the eye to notice. According to Paravina et al,^31^ 50% of observers accept a color variation at the acceptability threshold of ΔE00 = 1.8. In contrast, ΔE00 = 0.8 is used as the perceptibility threshold in half of the research on color change.

 Recent studies have examined the color stability of composite resins.^32-34^ According to the findings of these studies, prolonged immersion in staining drinks causes stains on surfaces.

 In particular, liquids with high tannin content, such as coffee and tea, cause stains on the surface of composite resin materials.^35^ In the present study, tea and coffee stained the composite resin samples at similar levels. Repolishing techniques have the potential to improve color stability and extend aesthetic life. These techniques can minimize color variations by reducing surface roughness and improving optical properties.^36^ In this study, tea, detox drink, cherry juice, coffee, and other artificial saliva solutions that people frequently consume daily were used as staining drinks. After 4 days of immersion, a significant color change was observed compared to the initial color. This change was primarily observed in the detox drink. A previous study showed that the type of detox drinks impacted the whiteness of the materials tested. The Detox Defence drink caused more staining than the other drinks, which was thought to be due to the yellow turmeric spice in the detox drink.^37^ The results of the above study are also compatible with the present study. In the study by Bagheri et al,^38^ cola did not stain as much as coffee and tea since it does not contain yellow dye, despite the fact that its low pH score impairs the surface integrity of composite materials. Regarding cherry juice, our study supported the results reported by Bagheri et al.

 Repolishing decreased color changes in bulk-fill composite resins after exposure to coffee, and two-stage polishing systems outperformed one-stage systems in this regard, according to a study on the effects of these processes on color change.^39^ In the present study, there was no discernible difference between the color changes recorded during staining and those measured in all groups following a two-stage repolishing procedure (*P* > 0.05). According to this result, it can be interpreted that repolishing can provide a color lightening (increase in L score) similar to the staining on the composite surfaces. According to a previous study, polishing and finishing systems significantly impact various universal composite resins, especially after immersion.^40^ Concerning eliminating stains from coffee-stained composite resins, polishing systems consisting of diamond particles outperformed those with silicon-carbide abrasive particles or aluminum oxide particles. In the present study, a polishing system consisting of diamond particles was used in agreement with the results of the study above.

 Previous studies have shown that the staining level decreases ΔL* scores for composite resin materials.^41-43^ In the present study, except for the detox drink and artificial saliva groups, ΔL_1_ scores decreased after staining.

 In the CIELab system, the b* coordinate is related to the colors yellow and blue. As the b* score increases, the color shifts toward yellow, and as the b* score decreases, the color shifts toward blue. Tekçe et al^42^ found a shift toward blue with distilled water exposure and toward yellow with black tea exposure in all composite resins. Poggio et al^43^ also found a significant increase in the Δb scores of composite resins exposed to coffee. In the present study, the mean b score decreased after the staining procedure and shifted toward blue in the artificial saliva group. In contrast, the b score increased and shifted toward yellow in the detox drink, tea, and coffee groups. While the b score approached the initial color after repolishing in the tea group, the statistical difference continued in the other groups.

 In the CIELab system, the a* coordinate is related to red and green colors. As the a* score increases, the color shifts toward red, and as the a* score decreases, the color shifts toward green. Tekçe et al^42^ assessed how three distinct beverages affected the color parameters of four distinct restorative materials. The Δa* score increased with each beverage in all materials. The Dyract XP showed the greatest shift in the Δa* score following Coca-Cola exposure. In the present study, in the tea, coffee, and cherry juice groups, the a* score increased with staining and decreased slightly with repolishing but did not return to the initial level. In the detox drink group, the a* score, which decreased significantly with staining, returned to the initial level after repolishing.

 Saliva, oral temperature, and tooth brushing are just some of the variables that may affect the staining potential of restorative materials in a clinical setting. The limitations of this study are that different polishing types and composite materials were not compared, and no aging process was applied to the specimens. It is recommended that additional in vitro and clinical studies be carried out to assess the materials’ color stability.

## Conclusion

 In this study, the ΔE scores obtained after staining and the ΔE scores obtained after polishing the monochromatic composite resin were closely similar. Therefore, it can be concluded that the composite specimens returned to their original color almost as much as they were colored after polishing. The clinician does not need to immediately replace a stained restoration. According to the findings of this study, repolishing of stained restorations is recommended before replacing them.

## Competing Interests

 No potential conflict of interest relevant to this study was reported.

## Ethical Approval

 This study was approved by the Kırıkkale University Non-interventional Research Ethics Committee on 16 Oct 2024 under meeting number 2024/12, with decision number 2024.01.21.
